# When the patient is the expert: measuring patient experience and satisfaction with care

**DOI:** 10.2471/BLT.18.225201

**Published:** 2019-05-28

**Authors:** Elysia Larson, Jigyasa Sharma, Meghan A Bohren, Özge Tunçalp

**Affiliations:** a Department of Biostatistics, Harvard T.H. Chan School of Public Health, 655 Huntington Ave. Building II, 4th floor, Boston, MA 02115, United States of America (USA).; b Department of Global Health and Population, Harvard T.H. Chan School of Public Health, Boston, USA.; c Centre for Health Equity, University of Melbourne School of Population and Global Health, Melbourne, Australia.; d Department of Reproductive Health and Research, World Health Organization, Geneva, Switzerland.

## Abstract

In 2018, three independent reports were published, emphasizing the need for attention to, and improvements in, quality of care to achieve effective universal health coverage. A key aspect of high quality health care and health systems is that they are person-centred, a characteristic that is at the same time intrinsically important (all individuals have the right to be treated with dignity and respect) and instrumentally important (person-centred care is associated with improved health-care utilization and health outcomes). Following calls to make 2019 a year of action, we provide guidance to policy-makers, researchers and implementers on how they can take on the task of measuring person-centred care. Theoretically, measures of person-centred care allow quality improvement efforts to be evaluated and ensure that health systems are accountable to those they aim to serve. However, in practice, the utility of these measures is limited by lack of clarity and precision in designing and by using measures for different aspects of person-centeredness. We discuss the distinction between two broad categories of measures of patient-centred care: patient experience and patient satisfaction. We frame our discussion of these measures around three key questions: (i) how will the results of this measure be used?; (ii) how will patient subjectivity be accounted for?; and (iii) is this measure validated or tested? By addressing these issues during the design phase, researchers will increase the usability of their measures.

## Résumé

En 2018, la publication de trois rapports indépendants soulignait la nécessité de prêter attention à la qualité des soins et de l'améliorer pour parvenir à une réelle couverture sanitaire universelle. L'un des aspects clés de la qualité des soins et des systèmes de santé est qu'ils soient centrés sur la personne, caractéristique qui revêt une importance à la fois intrinsèque (toutes les personnes ont le droit d'être traitées avec dignité et respect) et pratique (des soins centrés sur la personne sont associés à un plus grand recours aux soins et à de meilleurs résultats). Suite aux appels à l'action pour 2019, nous donnons des indications aux responsables politiques, aux chercheurs et aux personnes chargées de la mise en œuvre quant à la manière dont ils peuvent entreprendre de mesurer les soins centrés sur la personne. Théoriquement, les mesures des soins centrés sur la personne permettent d'évaluer les efforts visant à améliorer la qualité et garantissent la responsabilité des systèmes de santé vis-à-vis des patients. Or, dans la pratique, l'utilité de ces mesures est limitée par le manque de clarté et de précision de leur conception et par leur utilisation pour différents aspects de l'approche centrée sur la personne. Nous abordons ici la distinction entre deux grandes catégories de mesures des soins centrés sur le patient: l'expérience du patient et la satisfaction du patient. Notre discussion concernant ces mesures s'inscrit autour de trois questions clés: (i) comment les résultats de cette mesure seront-ils utilisés?; (ii) comment la subjectivité du patient sera-t-elle prise en compte?; (iii) cette mesure a-t-elle été validée ou testée? La prise en compte de ces points durant la phase de conception permettra aux chercheurs d’améliorer l'utilité de leurs mesures.

## Resumen

Se publicaron tres informes independientes en 2018, en los que se hacía hincapié en la necesidad de prestar atención a la calidad de la atención sanitaria y de mejorarla para lograr una cobertura sanitaria universal eficaz. Un aspecto fundamental de la atención sanitaria y los sistemas de salud de alta calidad es que están centrados en las personas, una característica que es al mismo tiempo intrínsecamente importante (todas las personas tienen derecho a ser tratadas con dignidad y respeto) e instrumentalmente importante (la atención centrada en las personas se asocia a una mejor utilización de la atención sanitaria y a mejores resultados en materia de salud). Tras los llamados para que el año 2019 sea un año de acción, proporcionamos orientación a los responsables de la formulación de políticas, investigadores y ejecutores sobre cómo pueden asumir la tarea de medir la atención centrada en las personas. Teóricamente, las medidas de atención centrada en la persona permiten evaluar los esfuerzos de mejora de la calidad y garantizar que los sistemas de salud rindan cuentas a aquellos a los que pretenden servir. Sin embargo, en la práctica, la utilidad de estas medidas se ve limitada por la falta de claridad y precisión en el diseño y el uso de medidas para diferentes aspectos de la atención centrada en la persona. Discutimos la distinción entre dos amplias categorías de medidas de atención centrada en el paciente: la experiencia del paciente y la satisfacción del paciente. Enmarcamos nuestro debate sobre estas medidas en torno a tres cuestiones clave: (i) ¿cómo se utilizarán los resultados de esta medida?; (ii) ¿cómo se contabilizará la subjetividad del paciente? y (iii) ¿se valida o se prueba esta medida? Al abordar estas cuestiones durante la fase de diseño, los investigadores aumentarán la utilidad de sus medidas.

## ملخص

تم في عام 2018 نشر ثلاثة تقارير مستقلة، تركز على الحاجة إلى الاهتمام بجودة الرعاية، والتحسينات فيها، بهدف تحقيق تغطية صحية عالمية فعالة. إن أحد الجوانب الرئيسية في الرعاية الصحية والنظم الصحية ذات الجودة العالية، هو ترتكز على الشخص ذاته، وهي خاصية ذات أهمية جوهرية في الوقت ذاته (يحق لكل الأفراد أن يتم التعامل معهم بكرامة واحترام)، وأهمية قانونية (ترتبط الرعاية التي تركز على الشخص ذاته بالاستخدام المُحسّن للرعاية الصحية والنتائج الصحية). بعد الدعوات لجعل 2019 عامًا من العمل، قمنا بتقديم التوجيه لواضعي السياسات، والباحثين، وجهات التنفيذ، حول كيفية قيامهم بمهمة قياس الرعاية التي ترتكز على الشخص. من الناحية النظرية، تسمح قياسات الرعاية التي ترتكز على الشخص بتقييم جهود تحسين الجودة، والتحقق من أن النظم الصحية تتمتع بالمساءلة تجاه من تهدف إلى خدمتهم. ومع ذلك، ووفقاً لإطار الممارسة، فإن فائدة هذه القياسات تتسم بالمحدودية نظراً للافتقار للوضوح والدقة في تصميم، ولاستخدام القياسات لجوانب مختلفة في التركيز على الشخص. نحن نناقش الفارق بين فئتين واسعتين من قياسات الرعاية التي ترتكز على المريض: تجربة المريض ومدى رضا المريض. نحن نركز في مناقشتنا لهذه القياسات على ثلاثة أسئلة رئيسية: (1) كيف سيتم استخدام نتائج هذا القياسات؟؛ و(2) كيف سيتم تبرير ذاتية المريض؟؛ و(3) هل تم التحقق من هذه القياسات أو اختبارها؟ من خلال التعامل مع هذه الأمور خلال مرحلة التصميم، سيزيد الباحثون من قابلية استخدام هذه التدابير الخاصة بهم.

## 摘要

2018 年发表的 3 份独立报告强调了关注并改善护理质量的需求，以实现有效的全民健康覆盖。高质量的医疗保健和卫生系统的关键考量之一是以人为本，此特点的重要性不仅体现在本质层面上（所有人都有权得到有尊严的对待和尊重），还体现在应用层面上（以人为本的护理服务与提高医疗护理利用和改善健康效果息息相关）。在响应“2019 行动年”的号召后，我们就如何开展衡量以人为本的护理服务为政策制定者、研究人员和实施人员提供指导。理论上来说，以人为本的护理服务可衡量将使得我们可以就质量改善方面的工作进行评估，并且确保医疗体系对其服务对象负有责任。然而实际上，在以人为本不同层面设计和实施措施缺乏透明度和精准度，这限制了此类措施的实用性。针对以患者为中心的护理措施，我们讨论了患者体验和患者满意度两大类间的区别。我们围绕以下三个关键问题对这些措施展开讨论: (i) 如何使用该措施的结果？(ii) 如何考虑患者的主观性？以及 (iii) 该措施是否经过检验或测试？在设计阶段解决此类问题，将有助于研究人员提高其措施的可用性。

## Резюме

В 2018 году были опубликованы три независимых отчета, которые призывали уделять особое внимание качеству лечения и всячески улучшать его, чтобы добиться эффективного охвата населения услугами здравоохранения. Ключевым аспектом высококачественных систем медико-санитарного обслуживания является их ориентированность на человека. Данная характеристика важна сама по себе (так как все люди имеют право на достойное и уважительное отношение к себе), а также имеет важное практическое значение (так как медицинское обслуживание, ориентированное на потребности человека, ассоциируется с лучшим использованием ресурсов здравоохранения и лучшими результатами для здоровья). Следуя задаче сделать 2019 год годом действий, мы обеспечиваем директивные органы, исследователей и исполнителей рекомендациями относительно того, как решать задачу измерения показателей ориентированного на человека медико-санитарного обслуживания. Теоретически наличие критериев измерения медико-санитарного обслуживания, ориентированного на потребности человека, позволяет оценивать усилия по совершенствованию качества и гарантирует подотчетность систем здравоохранения тем, кому они предназначены служить. Однако на практике функциональность таких критериев измерения ограничивается недостаточной четкостью и точностью разработки, а также тем, что для измерений используются различные аспекты ориентации на человека. Мы обсуждаем различие между двумя широкими категориями критериев измерения ориентированного на человека медико-санитарного обслуживания: личным опытом пациента и удовлетворенностью пациента лечением. Обсуждение построено вокруг трех наиболее существенных факторов: (i) как будут использоваться результаты этого измерения; (ii) как будет учитываться субъективный характер отношения пациента к лечению; (iii) проводились ли оценка достоверности или официальное подтверждение данного критерия измерения. Отвечая на эти вопросы на этапе разработки, исследователи повысят функциональность критериев измерения качества медицинского обслуживания.

## Introduction

In 2018, three independent reports^1^^–^^3^ propelled quality to the forefront of global conversations on health policy and practice. The reports were published by the United States National Academies of Sciences, Engineering, and Medicine;^1^ the World Health Organization, the Organisation for Economic Co-operation and Development and the World Bank;^2^ and the Lancet Global Health Commission on High-Quality Health Systems in the Sustainable Development Goals (SDG) era.^3^ All three reports emphasized that improvements to quality of health care are necessary to achieve effective universal health coverage, a central theme within SDG 3, that is, to ensure healthy lives and promote well-being for all at all ages.^4^ The reports defined quality of care as care that is effective in maintaining or improving health and is person-centred, meaning that it is “respectful of and responsive to individual preferences, needs, and values.”^1^

Person-centeredness is an essential aspect of quality for two reasons. First, it is intrinsically important because individuals have the right to be treated with dignity and respect when they are using health-care services. Second, it is instrumentally important as person-centred care is associated with improved health-care utilization and health outcomes.^5^ The focus on person-centred measures is not new; the Institute of Medicine’s landmark 2001 report on quality of care brought attention to what was then referred to as patient-centred care.^6^ Since then, many person-centred measures have been proposed in the research literature. In theory, the measures allow quality improvement efforts to be evaluated and health-care systems to be held accountable to those whom they aim to serve. However, in practice, these measures are easy to misuse, since they all rely on the patient’s report of their visit. The utility of the measures is thus limited by lack of clarity and precision in designing different types of measures.

In this paper, we discuss the important distinction between two broad categories of person-centred measures of quality of care: patient experience (the interactions that patients have with the health system) and patient satisfaction (patients’ evaluation of the care provided relative to their expectations). We provide positive examples from the maternal and child health literature to illustrate how these measures can be used.

## Using quality measures effectively

**Fig. 1:**
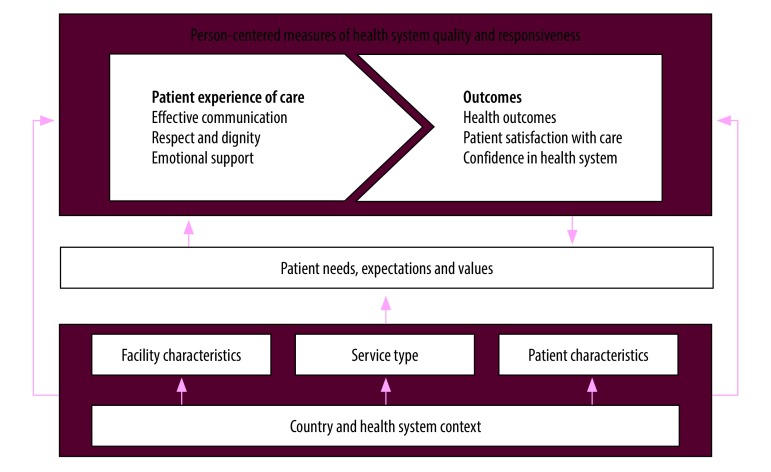
Framework for person-centred measures of health system quality and responsiveness

Sources: Based on Kruk et al.^3^ and Tunçalp et al.^7^

The first step is to define how person-centred measures of health relate to one another and then to discuss clear steps that researchers, policy-makers and implementers can take to ensure that the measures can be used effectively. Fig. 1 illustrates the inter-relationship between measures of patients’ experiences of and satisfaction with care. This scheme builds on the frameworks developed by the Lancet Global Health Commission^3^ and the World Health Organization vision for quality of care for pregnant women and newborns.^7^

Patient experience is a process indicator and reflects the interpersonal aspects of quality of care received. This indicator is broadly composed of three domains: effective communication; respect and dignity; and emotional support.^7^^,^^8^ These domains may be modified directly by factors, such as facility characteristics (e.g. number of patients seen, ratio of health-care providers to patients, availability of services and resources); patients’ characteristics (e.g. sociodemographic characteristics, clinical history, prior health care-seeking behaviour); and the type of service (e.g. preventive or non-emergency care versus emergency care). These modifiers themselves will depend on the country and health system. Alternatively, these modifiers can influence patients’ experiences more indirectly by shaping patients’ needs, expectations and values.

In contrast, patient satisfaction is an outcome measure of a patient’s experiences of care, along with health outcomes and confidence in the health system (Fig. 1), reflecting whether or not the care provided has met the patient’s needs and expectations.^3^ A patient’s needs and expectations are dynamic and may evolve depending on the care provided and the patient’s awareness of both facility-level (e.g. case fatality rates) and individual outcomes (e.g. health outcomes or patient satisfaction). Outcomes, including patient satisfaction, can both affect and be affected by patients’ needs, expectations and values. A patient’s experience of care may have a direct impact on the patient’s satisfaction, as well as an indirect impact through affecting the patient’s needs, expectations and values, which in turn affect satisfaction. Previous research has suggested that broader social factors, including patient characteristics, such as age and education, can explain variations in patients’ experiences of care, ability to evaluate the quality of care received, and satisfaction with care.^9^ Patient’s expectations and interpretations of their experiences of care are further shaped by the broader societal, community, and family contexts.

To produce evidence that can be acted on, we encourage researchers and implementers, (e.g. nongovernmental organizations delivering quality improvement programmes, local governments who manage care or private health-care providers) to consider three issues when using person-centred measures.^1^^–^^3^ First, because measures of patient experience and satisfaction are distinct, they represent different underlying constructs and are affected by different factors, choosing a measure based on how that measure will be used is essential. Second, because the reference standard for person-centred measures is the patient’s report, considering how subjectivity may play a role in the reporting is important. Third, we need to know whether the measures have been previously tested and validated.

## Choosing person-centred measures

### Defining the purpose

Person-centred measures are useful to policy-makers and implementers for guiding and evaluating quality improvement efforts and for holding the health system and its stakeholders accountable to the communities they serve. The choice of measures will depend on the purpose of the measurements, for example whether they will be used for improving quality of care or health-system accountability.

As process measures, patient experience measures may be sensitive to differences in quality care across different providers, institutions or time, and thus can be used to identify gaps or evaluate changes in quality resulting from interventions or policies.^10^ For example, in East Africa patient experience measures have been used to quantify types of disrespectful care during childbirth and inform targeted interventions towards improving care.^11^^,^^12^

Patient experience indicators are currently used to target quality improvement for maternal health care across nine countries within the Network for Improving Quality of Care for Maternal, Newborn and Child Health.^13^ One focus of the network is to improve support for women during labour and childbirth from a companion of her choice (such as a partner, sister or friend). Companionship in labour is associated with both improved patient experience, such as more positive experiences of childbirth, and better health and well-being outcomes, such as increased spontaneous vaginal birth, shorter duration of labour and higher 5-minute Apgar scores for the baby.^14^^,^^15^ By monitoring indicators of patient experience, such as the proportion of women wanting a labour companion compared to those who have one present, countries will be able to target areas in need of quality improvement interventions and evaluate the success of those interventions.

Measures of patient satisfaction can also be outcome indicators that reflect whether the care provided meets an individual’s needs and expectations. Satisfaction measures are useful for identifying areas of service provision that are important to individuals, or when aggregated for communities. However, the use of these measures requires caution, as changes in satisfaction level may be due to changes in quality of care or patient demand, values or expectations. Exploratory or qualitative research could help determine the underlying causes of changes in satisfaction. The role of expectations is discussed further in the next section.

Holding health systems and policy-makers accountable to the communities they serve is an instance where measures of both patient experience and satisfaction may be useful. A study conducted in government-managed primary care clinics in rural United Republic of Tanzania in 2018 assessed the ability of public feedback, through posters announcing facility performance on indicators of patient experience to hold health-care providers accountable for the quality of care they deliver.^16^ By using measures of patient experience, the study provided specific areas that health-care providers could target for improvement. Alternatively, if satisfaction measures are used for accountability, poor satisfaction scores might drive providers or policy-makers to identify aspects of services that are valuable to patients but where service provision is failing.

Person-centred care should be measured with a clear purpose. Patient experience measures can be used to evaluate quality of care, while satisfaction measures can track patients’ (or communities’) responses to care but not actual changes to care itself. While both measures can be used to hold health systems accountable, it must be clear whether the aim of accountability is to provide high quality care (patient experience) or be responsive to expectations of the population (patient satisfaction).

### Addressing subjectivity

The success of person-centred measures for quality improvement or accountability depends on how directly the indicators measure the underlying construct as intended. Understanding and assessing patients’ experiences and satisfaction, by definition, requires asking patients, but these self-reports will inherently introduce subjectivity. To address subjectivity when assessing person-centred measures, researchers must consider the phrasing of the questions, response choices and whether the questions account for patients’ expectations.

How questions are framed determines the degree of subjectivity of measures. Questions that ask patients to provide a direct report of what happened, as is the case for measures of experiences of care, tend to be less subjective than those that ask patients to evaluate or rate their experience, as in the case for all satisfaction measures (and some experience measures).^17^ Within the dimension of patient experience of communication, consider for example the following question, found in a patient experience survey in the United States of America: “Before giving you any new medicine, how often did hospital staff describe possible side effects in a way you could understand?”^18^ By including the phrase “in a way you could understand,” the question changes from asking patients to report on care that was provided (or not) to asking them to evaluate their experience of care. This inclusion makes the question more subjective, which is important if researchers want to understand if providers are communicating in an effective manner for patients across a diverse population. Patients have different needs, and health systems must be responsive and adaptive to these variations. Subjectivity is also important when considering whether results should be adjusted for case-mix, for example, age, health status or type of care, a common practice when looking at health outcomes. When patient characteristics are strongly associated with patient experience measures, adjustment may be useful if the goal is to compare across facilities.^19^ However, we believe that case-mix adjustment should not be used as a method to create more positive scores or dismiss lower scores, but rather to understand which populations may be having sub-optimal experiences of care and how their experiences can be improved.

Similarly, carefully weighing response options to questions is important. Choices such as “yes, always”, “yes, sometimes” and “no” are more objective than responses such as “excellent”, “good”, “fair” and “poor.” Whereas the former elicits a factual description, the latter relies on an individual’s perception. To interpret and act from these more subjective questions and response options, the evaluation may need to use vignettes, as described later, or obtain additional information on patient characteristics.^20^

Another example is the case of mistreatment of women during childbirth, such as being slapped or pinched. A woman’s report of mistreatment (objective measure) is likely to align with other objective measures, such as actual observations of mistreatment. Whereas her experiences of stigma and discrimination (subjective measure) may depend on her expectations of the health system, the provider or her lived experiences of discrimination (Bohren MA et al., University of Melbourne School of Population and Global Health, Australia, unpublished data, 2019). This difference is important to consider when observation is used as a method of data collection, because it is difficult for an independent observer to capture stigma and discrimination (Bohren MA et al., University of Melbourne School of Population and Global Health, Australia, unpublished data, 2019).^21^

Satisfaction with care is inherently shaped by an individual’s values, expectations and experiences, such as expecting to have a health-care provider who includes them in decision-making, and thus is a highly subjective measure requiring a nuanced approach to its interpretation. Patients’ expectations and values are affected both by factors that are related to the health system, for example availability of care, and by factors outside of the health system, such as an individual’s social identity.^22^^,^^23^ Since dynamic factors can affect an individual’s expectations for care, and in turn her or his satisfaction with care and utilization of services, we must understand expectations and how these may change.^9^

Expectations can be assessed qualitatively or quantitatively. Qualitative research is useful to elucidate ideas that may be previously unknown to the researcher or to explore a person’s values and preferences. Anchoring vignettes can be useful; these are hypothetical situations or stories that the respondents evaluate, perhaps by rating their satisfaction with the care described. Such vignettes provide an opportunity to quantify an individual’s expectations.^24^ Since all respondents evaluate the same situation, differences between respondents’ judgements can be considered as due to different expectations, thus allowing for adjustment of their rating of their own care. By further assessing factors that affect satisfaction, including individuals’ expectations, values and awareness of care available to them, we can draw more informed conclusions about why satisfaction may differ or change across time and populations.

### Validating and testing

For person-centred measures, the reference standard is patients’ self-reporting. This type of reporting makes validation and testing of measures and scales different from many health outcomes that have an objective reference standard, such as using blood pressure to diagnose hypertension. However, self-reporting does not exempt researchers from validating, or at least testing, their measures. Using validated measures can help researchers address the issue of subjectivity. We suggest focusing efforts on construct and content validity. 

First, researchers should consider how well their measures reflect an established model or theory (construct validity). It is important to consider if these general constructs have been measured or tested before and if previously validated measures are available.

Second, researchers should assess how well the measure represents the probable range of patient experience or satisfaction (content validity). This validation should occur in the context where the research is taking place, considering both the type of care (e.g. primary care or hospital) and geographical or cultural context. Qualitative research with the population of interest is appropriate for content validation; for example, conducting focus group discussions with patients to understand if the proposed tools are measuring the experiences or satisfaction.

Finally, researchers should consider how a measure performs across populations. Not all measures need to be reliable across populations and settings, but this is an important consideration when assessing the generalizability of findings. When reliability across settings is important, for example, to allow data to be compared across populations, using existing measures is beneficial. Acknowledging this need, the World Health Organization is currently developing a guidance document on validation of indicators for monitoring maternal and newborn health.

A demonstration of these steps has been shown for validation of a person-centred maternity care index.^25^^,^^26^ Researchers used a literature review and interviews with experts to assess content validity and then interviewers administered surveys with postpartum women in both India and Kenya to assess the criterion validity and reliability of the index. This validation work has resulted in a 30-item scale that can be used to facilitate measurements of and eventually improvements to person-centred maternity care within populations that are similar to those for which the scale was validated.

Similarly, a multimethod, multistep approach has been used for developing tools to measure the mistreatment of women during childbirth.^27^ Based on a global systematic review^28^ and primary qualitative research in four countries,^27^ the researchers developed two measurement tools: observations of labour and childbirth, and a postpartum survey with women. The researchers conducted validity testing with maternal health experts and women who recently gave birth, and adjusted the tools based on the responses of the population of interest.^27^

Despite this focus on quantitative measurement of person-centred care, qualitative methods, including interviews and focus group discussions, can help provide more in-depth and descriptive information about a patient’s experience.^29^ For example, in-depth interviews, where patients are asked to detail their experiences with a health service, are a useful tool for designing better services.^29^ Qualitative methods can also be used to help validate and enhance information gained from quantitative measures.

## Conclusions

We are currently in an age of renewed attention to quality, a necessary component of care for universal health coverage to be effective in improving peoples’ lives.^30^ Measurement of person-centred care is a key step towards ensuring accountability and action and quality of care improvement.^3^ When measures do not have a clear purpose or are incorrectly specified or interpreted, they risk conveying an inaccurate and unreliable assessment of quality of care. This inappropriate use of measures can waste time and resources, both in the initial collection of data and in initiatives and policies resulting from poor measurement. We have outlined questions that can guide the generation of clear, actionable evidence. Clarity in thinking and precision in using person-centred measures will advance the science and practice of delivering respectful and effective health care.
